# Development and validation of a new scoring system for prognostic prediction of community-acquired pneumonia in older adults

**DOI:** 10.1038/s41598-021-03440-3

**Published:** 2021-12-13

**Authors:** Masahiro Shirata, Isao Ito, Tadashi Ishida, Hiromasa Tachibana, Naoya Tanabe, Satoshi Konishi, Issei Oi, Nobuyoshi Hamao, Kensuke Nishioka, Hisako Matsumoto, Yoshiro Yasutomo, Seizo Kadowaki, Hisashi Ohnishi, Hiromi Tomioka, Takashi Nishimura, Yoshinori Hasegawa, Atsushi Nakagawa, Toyohiro Hirai

**Affiliations:** 1 Department of Respiratory Medicine, Graduate School of Medicine, Kyoto University, 54 Shogoin-kawaharacho, Sakyo, Kyoto, 606-8507 Japan; 2 Department of Respiratory Medicine, Ohara Healthcare Foundation, Kurashiki Central Hospital, Okayama, Japan; 3 Department of Internal Medicine, Ono Municipal Hospital, Hyogo, Japan; 4 Department of Respiratory Medicine, Akashi Medical Center, Hyogo, Japan; 5 Department of Respiratory Medicine, Kobe City Medical Center West Hospital, Hyogo, Japan; 6 Department of Respiratory Medicine, Kyoto Katsura Hospital, Kyoto, Japan; 7 Department of Respiratory Medicine, Osaka Saiseikai Nakatsu Hospital, Osaka, Japan; 8 Department of Respiratory Medicine, Japanese Red Cross Wakayama Medical Center, Wakayama, Japan

**Keywords:** Bacterial infection, Prognostic markers

## Abstract

The discriminative power of CURB-65 for mortality in community-acquired pneumonia (CAP) is suspected to decrease with age. However, a useful prognostic prediction model for older patients with CAP has not been established. This study aimed to develop and validate a new scoring system for predicting mortality in older patients with CAP. We recruited two prospective cohorts including patients aged ≥ 65 years and hospitalized with CAP. In the derivation (n = 872) and validation cohorts (n = 1,158), the average age was 82.0 and 80.6 years and the 30-day mortality rate was 7.6% (n = 66) and 7.4% (n = 86), respectively. A new scoring system was developed based on factors associated with 30-day mortality, identified by multivariate analysis in the derivation cohort. This scoring system named CHUBA comprised five variables: confusion, hypoxemia (SpO_2_ ≤ 90% or PaO_2_ ≤ 60 mmHg), blood urea nitrogen ≥ 30 mg/dL, bedridden state, and serum albumin level ≤ 3.0 g/dL. With regard to 30-day mortality, the area under the receiver operating characteristic curve for CURB-65 and CHUBA was 0.672 (95% confidence interval, 0.607–0.732) and 0.809 (95% confidence interval, 0.751–0.856; *P* < 0.001), respectively. The effectiveness of CHUBA was statistically confirmed in the external validation cohort. In conclusion, a simpler novel scoring system, CHUBA, was established for predicting mortality in older patients with CAP.

## Introduction

Community-acquired pneumonia (CAP) is a leading cause of morbidity and mortality among infectious diseases worldwide, particularly in older adults, in whom it also has a high incidence rate^1^. With an increasing elderly population in several countries, appropriate management of CAP is gaining importance^2^.

In the treatment of CAP, initial assessment of disease severity is critical as early stratification of patients according to mortality risk may help decide the therapeutic approach, including the site of care and the intensity of diagnostic testing and antibiotic therapy^1^. To date, many severity assessment tools have been proposed as prognostic prediction models for CAP^3^. Among them, the pneumonia severity index (PSI) and CURB-65 [i.e., confusion, urea > 7 mmol/L, respiratory rate ≥ 30 breaths/min, blood pressure (systolic < 90 mmHg or diastolic ≤ 60 mmHg), and age ≥ 65 years] are the most commonly used globally as well-validated models^4,5^. PSI has been shown to exhibit a high discriminatory power for mortality from CAP^6^. However, this prediction model has limited clinical application in a busy hospital setting as it consists of 20 variables and comprises complicated calculations^7,8^. Conversely, CURB-65 is easy to use, and several validation studies have demonstrated that its discriminatory ability for mortality from CAP is almost comparable to that of PSI^9–11^. However, there has been concern that the performance of CURB-65 significantly decreases with increasing age, compared to that of PSI^12–14^. Several new scoring systems, such as CORB75 (confusion, oxygen saturation, respiratory rate, systolic blood pressure, age ≥ 75 years) and UBMo index (the urea multiplied by the N-terminal-pro-brain natriuretic peptide plasmatic rate, divided by the monocyte count) have been devised to improve the performance of prognostic prediction for CAP in the older population^15,16^. However, a useful prognostic prediction model for these patients based on a prospective and well-validated cohort has not yet been established^12,14,17–19^.

Since Japanese people have one of the highest life expectancies in the world and most patients hospitalized with pneumonia are older adults, we thought a model for prognostic prediction of CAP in older adults would be beneficial to the aging population. Thus, we conducted this study with the aim of identifying the risk factors associated with 30-day mortality in patients aged ≥ 65 years admitted with CAP, developing a simpler novel scoring system for predicting the prognosis of CAP in these older populations, and evaluating its effectiveness with an external validation cohort.

## Methods

### Study design

This prospective observational study enrolled consecutive patients admitted with a diagnosis of CAP. The inclusion criteria were community-acquired pneumonia, defined as pneumonia acquired outside of the hospital, in all patients aged ≥ 65 years who were admitted to the hospital. Pneumonia was diagnosed as described in a previous publication that included a few of the patients from this study^20^, based on the radiological appearance of new and/or progressive pulmonary infiltrates and the presence of at least two of the following conditions: cough, sputum or change in sputum characteristic (e.g., increased volume and/or purulence), dyspnea, tachypnea, abnormal breathing sounds, pleuritic chest pain, auscultatory findings on chest examination consistent with lung infiltrates, documented axillary body temperature of ≥ 37.5 °C within the past 24 h, chills and/or shivers, general malaise, and a white blood cell count ≥ 10,000/µL or < 3,000/µL. The exclusion criteria were hospital-acquired pneumonia, terminal stage of active cancers, and other pulmonary infiltrative diseases, such as radiation pneumonitis, organizing pneumonia, drug-induced pneumonia, active lung cancer, tuberculosis, fungal infection, and empyema.

The derivation cohort included patients with CAP who were admitted to Ono Municipal Hospital between September 1, 2003, and December 31, 2012. A new prognostic scoring system for older adults ≥ 65 years was developed from the derivation cohort. The external validation cohort included patients with CAP admitted to Ohara Healthcare Foundation, Kurashiki Central Hospital, between January 1, 2007 and December 31, 2010 and to six other Japanese hospitals, covering several geographical areas, between January 1, 2010 and December 31, 2010.

This study was conducted in accordance with the Declaration of Helsinki and its later amendments and was approved by the institutional review board of the participating institutions. The requirement for written informed consent was waived, and oral consent was obtained from all patients prior to enrolment.

### Data collection and study outcome

The following variables were assessed: age, sex, pre-hospital residence, bedridden state prior to admission, comorbidities, and initial vital signs upon arrival to the hospital. Laboratory data were analyzed using blood samples collected within 24 h before or after admission. Bedridden state was defined as being totally confined to the bed or chair with loss of self-care abilities.

The severity of pneumonia on admission was assessed using PSI and/or CURB-65^4,5^; all patients were classified into PSI class I–V and/or assigned to six strata based on the five variables of CURB-65, as defined in the original studies^4,5^. The PSI could not be assessed in the validation cohort because the respiratory status in several patients was assessed using SpO_2_ rather than arterial blood gas analysis. The outcomes of interest in this study were all-cause mortality within 30 days of admission (30-day mortality) and during hospitalization (in-hospital mortality).

### Statistical analysis

A complete-case analysis was performed as there were few missing values for each variable in these prospective cohorts. Categorical variables were presented as numbers (percentages) and compared using Pearson’s Chi-square test or Fisher’s exact test. Continuous variables were presented as mean ± standard deviation, and Student’s t-test for normally distributed data or the Mann–Whitney *U* test for non-normally distributed data were used for comparisons.

The least absolute shrinkage and selection operator (LASSO) regression, which can minimize the potential collinearity and over-fitting of variables, was applied to evaluate candidates for constituent variables in the new prognostic scoring system. To convert a continuous variable to a nominal variable, the optimum cut-off values of continuous variables were determined as those that gave the maximum value of the Youden index, defined as [(sensitivity + specificity) − 1]. Regarding these values, an approximate value that might be easy-to-use in clinical settings was adopted as the cut-off value. Subsequently, variables identified by LASSO regression analysis were entered into logistic regression models and all statistically significant variables were included in the new scoring system.

Using the receiver operating characteristic (ROC) curve analysis, the area under ROCs (AUROCs) with 95% confidence intervals were compared to evaluate the discriminatory power of the new scoring system, PSI and/or CURB-65 for predicting mortality^21,22^. Patients from the derivation and validation cohorts were stratified into risk groups according to the new scoring system, and survival curves in each group were depicted using the log-rank test with the Kaplan–Meier method.

All tests were two-sided, and *P* < 0.05 was considered statistically significant. All statistical analyses were conducted using JMP Pro14.0.0 (SAS Institute Inc., Cary, NC, USA).

## Results

### Patient characteristics

Among 1,157 patients in the derivation cohort, 916 patients with CAP were ≥ 65 years old, 44 of whom were excluded because of missing data pertaining to at least one variable. Among 1,418 patients in the validation cohort, 1,180 patients were ≥ 65 years old, 22 of whom were excluded because of missing data and two because of unknown clinical outcomes. Ultimately, we included 872 patients in the derivation cohort and 1,158 patients in the validation cohort.

The baseline characteristics and main clinical outcomes of the patients across each cohort are summarized in Table 1. The average age of patients in the derivation and validation cohorts was 82.0 and 80.6 years, respectively. Chronic pulmonary disease was the most prevalent comorbidity in both cohorts. The 30-day mortality was almost the same between the two cohorts (7.6% vs. 7.4%, *P* = 0.904).

**Table 1:** Baseline characteristics of the patients.

	Derivation cohort (n = 872)	Validation cohort (n = 1,158)	*P* value
**Demographics**			
Age (y)	82.0 ± 8.1	80.6 ± 8.2	< 0.001
Male	461 (52.9)	737 (63.6)	< 0.001
Female	411 (47.1)	421 (36.4)	< 0.001
Bedridden state	192 (22.0)	255 (22.0)	0.999
Long-term care facility resident	213 (24.4)	183 (15.8)	< 0.001
**Comorbidity**			
Malignant disease	27 (3.1)	129 (11.1)	< 0.001
Cerebrovascular disorder	265 (30.4)	321 (27.7)	0.189
Chronic pulmonary disease	277 (31.8)	488 (42.1)	< 0.001
Chronic heart disease	152 (17.4)	338 (29.2)	< 0.001
Chronic liver disease	33 (3.8)	50 (4.3)	0.547
Chronic kidney disease	34 (3.9)	107 (9.2)	< 0.001
Diabetes mellitus	140 (16.1)	222 (19.2)	0.068
**Clinical outcome**			
Mechanical ventilation	23 (2.6)	73 (6.3)	< 0.001
Vasopressor infusion	9 (1.0)	40 (3.5)	< 0.001
Duration of hospitalization (days)	27.7 ± 29.2	18.5 ± 30.0	< 0.001
30-day mortality	66 (7.6)	86 (7.4)	0.904
In-hospital mortality	109 (12.5)	106 (9.2)	0.016

Data are presented as mean ± standard deviation or n (%).

### Risk factors associated with 30-day mortality

The results of the univariate analysis regarding risk factors associated with 30-day mortality in the derivation cohort are shown in Table 2. Several factors were associated with poor prognosis, including demographic features such as age, bedridden state, residence in long-term care facility (LTCF), comorbidity of cerebrovascular disease and chronic kidney disease, vital signs upon admission including consciousness, blood pressure, respiratory condition, and laboratory findings of anemia, thrombocytopenia, hypoalbuminemia, azotemia, and hyponatremia.

**Table 2:** Univariate analysis and LASSO regression of prognostic factors associated with 30-day mortality.

	Survivors (n = 806)	Non-survivors (n = 66)	Univariate analysis		LASSO regression	
			OR (95% CI)	*P* value	EC (95% CI)	*P* value
**Demographics**						
Age (y)	81.7 ± 8.1	86.4 ± 6.0	–	< 0.001	–0.030 (− 0.062–0.001)	0.061
Male	419 (52.0)	42 (63.6)	1.62 (0.961–2.72)	0.066	0.370 (− 0.155–0.896)	0.167
Bedridden state	159 (19.7)	33 (50.0)	4.07 (2.44–6.80)	< 0.001	0.592 (0.019–1.16)	0.043
Long-term care facility resident	189 (23.5)	24 (36.4)	1.87 (1.10–3.16)	0.024	0 (0–0)	1.000
**Comorbidity**						
Malignant disease	23 (2.9)	4 (6.1)	2.20 (0.736–6.55)	0.141	0 (0–0)	1.000
Cerebrovascular disorder	236 (29.3)	29 (43.9)	1.89 (1.14–3.15)	0.016	0 (0–0)	1.000
Chronic pulmonary disease	258 (32.0)	19 (28.8)	0.859 (0.494–1.49)	0.586	0 (0–0)	1.000
Chronic heart disease	139 (17.3)	13 (19.7)	1.18 (0.625–2.22)	0.619	0 (0–0)	1.000
Chronic liver disease	29 (3.6)	4 (6.1)	1.73 (0.589–5.07)	0.306	0 (0–0)	1.000
Chronic kidney disease	27 (3.4)	7 (10.6)	3.42 (1.43–8.19)	0.013	0.892 (− 0.224–2.01)	0.117
Diabetes mellitus	132 (16.4)	8 (12.1)	0.704 (0.329–1.51)	0.349	0 (0–0)	1.000
**Vital signs**						
Confusion	95 (11.8)	24 (36.4)	4.28 (2.48–7.38)	< 0.001	0.737 (0.151–1.32)	0.014
Systolic blood pressure (mmHg)	132.7 ± 25.3	123.2 ± 31.0	–	0.004	0–0	1.000
Diastolic blood pressure (mmHg)	73.2 ± 14.8	69.7 ± 16.8	–	0.066	0–0	1.000
Heart rate (beats/min)	89.2 ± 17.6	92.0 ± 18.4	–	0.219	0–0	1.000
Respiratory rate ≥ 30 breath/min	93 (11.5)	12 (18.2)	1.70 (0.879–3.30)	0.132	0–0	1.000
Hypoxemia^#^	304 (37.7)	44 (66.7)	3.30 (1.94–5.62)	< 0.001	0.622 (0.085–1.16)	0.023
**Laboratory examinations**						
WBC (× 10^3^/µl)	11.3 ± 5.3	11.4 ± 7.3	–	0.846	0–0	1.000
Hct (%)	36.1 ± 5.3	33.8 ± 5.4	–	0.001	0–0	1.000
PLT (× 10^4^/µl)	22.0 ± 8.2	19.7 ± 8.5	–	0.030	0.006 (-0.026–0.039)	0.704
Alb (g/dl)	3.47 ± 0.50	2.88 ± 0.53	–	< 0.001	1.33 (0.840–1.81)	< 0.001
BUN (mg/dl)	22.5 ± 13.4	34.2 ± 29.4	–	< 0.001	-0.012 (-0.023–0.001)	0.034
Na (mEq/l)	138.4 ± 4.9	136.8 ± 9.5	–	0.020	0.005 (-0.035–0.045)	0.806
Glu (mg/dl)	142.6 ± 57.3	144.5 ± 61.8	–	0.803	0–0	1.000
CRP (mg/dl)	10.4 ± 8.1	11.3 ± 8.1	–	0.400	0–0	1.000

Data are presented as mean ± standard deviation or n (%).

^#^Hypoxemia: SpO_2_ ≤ 90% or PaO_2_ ≤ 60 mmHg.

OR, odds ratio; CI, confidence interval; EC, estimated coefficients; WBC, white blood count; Hct, hematocrit; PLT, platelet; Alb, albumin; BUN, blood urea nitrogen; Na, sodium; Glu, glucose; CRP, C-reactive protein.

On LASSO regression including 25 variables, the following five candidate variables were associated with 30-day mortality: bedridden state, confusion, hypoxemia, azotemia, and hypoalbuminemia (Table 2). To convert continuous variables to nominal variables, the cut-off values were set to 30 mg/dL for blood urea nitrogen (BUN) and 3.0 g/dL for serum albumin. BUN and serum albumin for those values that maximized the Youden index were 26.7 mg/dL and 3.2 g/dL, respectively. A logistic regression model including these five variables indicated that they were all independently statistically significant predictors of 30-day mortality (Table 3).

**Table 3:** Multivariate logistic regression analysis of prognostic factors associated with 30-day mortality.

Variables	OR (95% CI)	*P* value
Bedridden state	2.64 (1.52–4.58)	< 0.001
Confusion	2.54 (1.40–4.62)	0.003
Hypoxemia^#^	2.27 (1.29–4.01)	0.004
BUN ≥ 30 mg/dl	2.82 (1.62–4.91)	< 0.001
Alb ≤ 3.0 g/dl	3.03 (1.74–5.27)	< 0.001

Data are presented as n (%).

^#^Hypoxemia: SpO_2_ ≤ 90% or PaO_2_ ≤ 60 mmHg.

OR, odds ratio; CI, confidence interval; Alb, albumin; BUN, blood urea nitrogen.

### Development of a new scoring system

A new scoring system for prognostic prediction of CAP in older adults, named CHUBA, (confusion, hypoxemia, urea, bedridden, albumin) was developed based on logistic regression analysis. Each variable was equally allocated one point since the odds ratios were almost equivalent. By adding up the points, a score that could range between 0 and 5 was allocated to each patient.

Table 4 shows the 30-day and in-hospital mortality according to each score of the PSI and/or CURB-65, and CHUBA. In all models, a positive correlation was observed between each score and mortality. The sensitivity, specificity, positive predictive value, negative predictive value, and Youden index of 30-day and in-hospital mortality at different cut-off values for each model are presented in Table 5A. The maximum values of the Youden index for CURB-65, PSI, and CHUBA were 0.242, 0.438, and 0.462, respectively, in predicting 30-day mortality; and 0.234, 0.406, and 0.453, respectively, in predicting in-hospital mortality, suggesting that the discriminative performance of the new system was superior to that of CURB-65, and slightly outperformed PSI.

**Table 4:** Comparison of conventional and new prognostic scoring in the derivation and validation cohorts.

	Score	Derivation cohort			Validation cohort		
		n	30-day mortality	In-hospital mortality	n	30-day mortality	In-hospital mortality
CURB-65	0	0	–	–	0	–	–
	1	314	9 (2.9)	17 (5.4)	329	8 (2.4)	11 (3.3)
	2	357	28 (7.8)	45 (12.6)	437	23 (5.3)	29 (6.6)
	3	155	19 (12.3)	31 (20.0)	278	31 (11.2)	38 (13.7)
	4	42	8 (19.0)	14 (33.3)	96	18 (18.8)	21 (21.9)
	5	4	2 (50.0)	2 (50.0)	18	6 (33.3)	7 (38.9)
PSI class	I	1	0 (0.0)	0 (0.0)			
	II	67	0 (0.0)	0 (0.0)			
	III	223	1 (0.4)	2 (0.9)			
	IV	419	26 (6.2)	48 (11.5)			
	V	162	39 (24.1)	59 (36.4)			
CHUBA	0	312	3 (1.0)	5 (1.6)	204	1 (0.5)	1 (0.5)
	1	258	12 (4.7)	23 (8.9)	405	6 (1.5)	11 (2.7)
	2	171	14 (8.2)	27 (15.8)	293	24 (8.2)	30 (10.2)
	3	93	20 (21.5)	32 (34.4)	164	30 (18.3)	37 (22.6)
	4	33	14 (42.4)	18 (54.5)	81	23 (28.4)	25 (30.9)
	5	5	3 (60.0)	4 (80.0)	11	2 (18.2)	2 (18.2)
Total population		872	66 (7.6)	109 (12.5)	1,158	86 (7.4)	106 (9.2)

Data are presented as n (%).

CURB-65, confusion, urea, respiratory rate, blood pressure, age ≥ 65; PSI, pneumonia severity index; CHUBA, confusion, hypoxemia, urea, bedridden, albumin.

**Table 5:** Operating characteristics of different prediction rules for 30-day and in-hospital mortality.

Model	Score	30-day mortality					In-hospital mortality				
		Sensitivity	Specificity	PPV	NPV	Youden Index	Sensitivity	Specificity	PPV	NPV	Youden Index
**(A) Derivation cohort**											
CURB-65	≥ 1	100.0	0.0	7.6	NA	0	100.0	0.0	12.5	NA	0
	≥ 2	86.4	37.8	10.2	97.1	0.242	84.4	39.0	16.5	94.6	0.234
	≥ 3	43.9	78.7	14.4	94.5	0.226	43.1	79.8	23.4	90.8	0.229
	≥ 4	15.2	95.5	21.7	93.2	0.107	14.7	96.1	34.8	88.7	0.108
	5	3.0	99.8	50.0	92.6	0.028	1.8	99.7	50.0	87.7	0.015
PSI class	≥ II	100.0	0.1	7.6	100.0	0.001	100.0	0.1	12.5	100.0	0.001
	≥ III	100.0	8.4	8.2	100.0	0.084	100.0	8.9	13.6	100.0	0.089
	≥ IV	98.5	36.0	11.2	99.7	0.345	98.2	37.9	18.4	99.3	0.361
	V	59.1	84.7	24.1	96.2	0.438	54.1	86.5	36.4	93.0	0.406
CHUBA	≥ 1	95.5	38.3	11.3	99.0	0.338	95.4	40.2	18.6	98.4	0.356
	≥ 2	77.3	68.9	16.9	97.4	0.462	74.3	71.0	26.8	95.1	0.453
	≥ 3	56.1	88.3	28.2	96.1	0.444	49.5	90.0	41.2	92.6	0.395
	≥ 4	25.8	97.4	44.7	94.1	0.232	20.2	97.9	57.9	89.6	0.181
	5	4.6	99.8	60.0	92.7	0.044	3.7	99.9	80.0	87.9	0.036
**(B) Validation cohort**											
CURB-65	≥ 1	100.0	0.0	7.4	NA	0	100.0	0.0	9.2	NA	0
	≥ 2	90.7	29.9	9.4	97.6	0.206	89.6	30.2	11.5	96.7	0.198
	≥ 3	64.0	68.6	14.0	96.0	0.326	62.3	69.0	16.8	94.8	0.313
	≥ 4	27.9	91.6	21.1	94.1	0.195	26.4	91.8	24.6	92.5	0.182
	5	7.0	98.9	33.3	93.0	0.059	6.6	99.0	38.9	91.3	0.056
CHUBA	≥ 1	98.8	18.9	8.9	99.5	0.177	99.1	19.3	11.0	99.5	0.184
	≥ 2	91.9	56.2	14.4	98.9	0.481	88.7	56.8	17.1	98.0	0.455
	≥ 3	64.0	81.3	21.5	96.6	0.453	60.4	81.8	25.0	95.3	0.422
	≥ 4	29.1	93.8	27.2	94.3	0.229	25.5	93.8	29.3	92.6	0.193
	5	2.3	99.2	18.2	92.7	0.015	1.9	99.1	18.2	90.9	0.010

Data are presented as percentage or n.

CURB-65, confusion, urea, respiratory rate, blood pressure, age ≥ 65; PSI, pneumonia severity index; CHUBA, confusion, hypoxemia, urea, bedridden, albumin; PPV, positive predictive value; NPV, negative predictive value; NA, not applicable.

**Figure 1:**
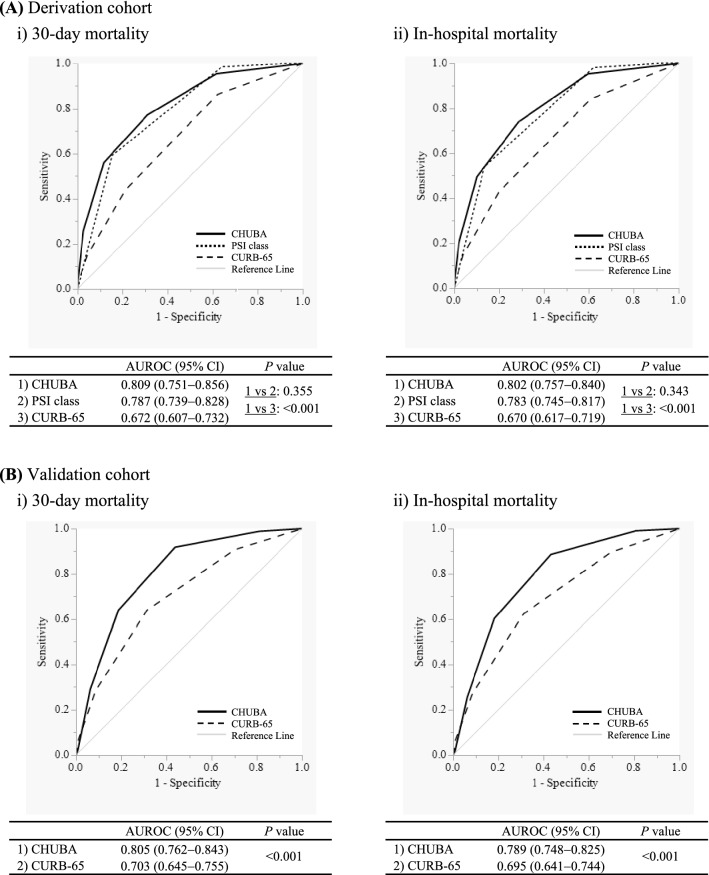
Comparisons of the discriminatory power of CURB-65 (confusion, urea, respiratory rate, blood pressure, age ≥ 65), pneumonia severity index (PSI) class, and CHUBA (confusion, hypoxemia, urea, bedridden, albumin) for predicting mortality among older patients hospitalized with a diagnosis of community-acquired pneumonia. (**A**) In the derivation cohort, the areas under the receiver operating characteristic (AUROCs) of CHUBA for prediction of both 30-day mortality and in-hospital mortality were significantly higher than those of CURB-65 (both *P* < 0.001) and were statistically equivalent to those of PSI class (*P* = 0.355 and *P* = 0.343, respectively). (**B**) In the validation cohort, AUROCs of CHUBA for prediction of both 30-day mortality and in-hospital mortality were significantly higher than those of CURB-65 (both *P* < 0.001).

The discriminatory ability of each scoring system for predicting the 30-day and in-hospital mortality was compared using the ROC curve (Fig. 1A). CHUBA had a discriminatory power superior to that of CURB-65 in predicting 30-day mortality (*P* < 0.001). CHUBA also performed well when predicting in-hospital mortality (*P* < 0.001). The discriminative power of CHUBA was statistically equivalent to that of PSI in predicting 30-day mortality (*P* = 0.355) and in-hospital mortality (*P* = 0.343). The superiority of CHUBA to CURB-65 was also maintained in two age-class subgroups: older (aged 65–84 years) and the very old individuals (age ≥ 85 years) (Supplementary Fig. 1A and Supplementary Fig. 1B).

In this study, age did not show a statistically significant association with the prognosis of CAP on multivariate analysis (*P* = 0.061), though it has been proposed as a prognostic factor for CAP in many previous studies^4,5,23^. Therefore, we also developed another prognostic prediction model named CHUBA-80, which included age as a variable. In this model, one point was allocated for age ≥ 80 years, based on the cut-off value that maximized the Youden index. However, it showed no significant improvement in the discriminatory power for 30-day and in-hospital mortality when compared with CHUBA (*P* = 0.200 and 0.896, respectively; Supplementary Fig. 2A and Supplementary Fig. 2B).

### Validation of the new scoring system

The performance of CHUBA compared to that of CURB-65 was evaluated using an external validation cohort. In this cohort, PSI had to be excluded from the comparison, as noted in the Methods section. There was an almost positive correlation between each score and mortality in CURB-65 and CHUBA (Table 4). The maximum Youden indices for predicting 30-day and in-hospital mortality were higher in CHUBA than in CURB-65 (0.481 vs. 0.326, and 0.455 vs. 0.313, respectively; Table 5B). Figure 1B shows that the AUROC of CHUBA in predicting 30-day mortality was significantly greater than that of CURB-65 (*P* < 0.001). This statistically significant difference in AUROC was also preserved in the prediction of in-hospital mortality (*P* < 0.001). Additionally, the effectiveness of CHUBA was observed in predicting 30-day mortality and in-hospital mortality in both age-class subgroups (Supplementary Fig. 3A and Supplementary Fig. 3B).

In summary, the findings obtained from the validation cohort were substantially in line with those of the derivation cohort, demonstrating the superiority of the discriminative ability of CHUBA over that of CURB-65.

### Survival curves of each group stratified by mortality risk

**Figure 2:**
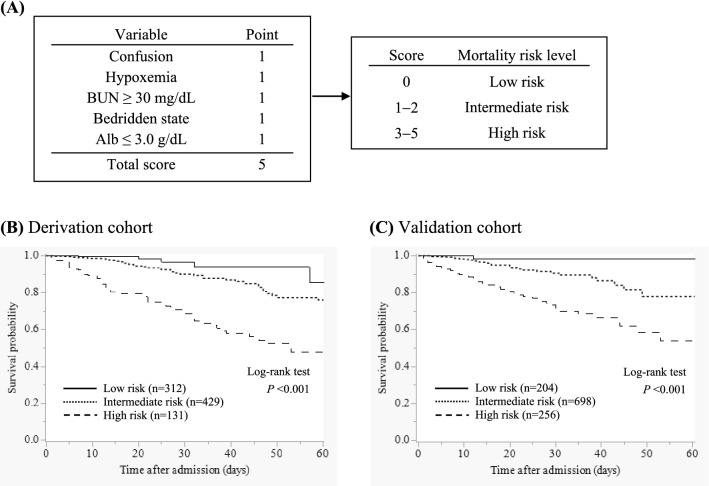
A new scoring system for prognostic prediction of community-acquired pneumonia in older patients and mortality risk stratification. (**A**) CHUBA (confusion, hypoxemia, urea, bedridden, albumin) was developed as a new scoring system, each variable of which was evenly allocated one point. All patients were stratified into three risk groups according to the CHUBA score. BUN, blood urea nitrogen; Alb, albumin. (**B**, **C**) Kaplan–Meier analysis of survival probability, stratified into low-risk, intermediate-risk, and high-risk groups according to the CHUBA score in the derivation and validation cohorts. The log-rank test indicates a significant difference between the survival curves in both cohorts (both *P* < 0.001).

All patients were stratified into three groups according to the CHUBA scores (score 0, low risk; score 1–2, intermediate risk; score 3–5, high risk) (Fig. 2A). Thereafter, the survival curves in each risk group of the derivation and validation cohorts were depicted using the Kaplan–Meier method (Fig. 2B and C), which showed a statistically significant difference in the survival rates between the risk groups in both cohorts (both *P* < 0.001).

## Discussion

In this study, for the purpose of prediction of mortality in patients aged ≥ 65 years hospitalized with CAP, we established a new scoring system consisting of only five variables that can be easily evaluated prior to admission. To the best of our knowledge, this is the first scoring system for predicting mortality in older patients with CAP, developed with a prospective cohort and validated with an external cohort.

It has been reported that aging reduces the discriminatory ability of CURB-65, a conventional and well-established scoring system to predict mortality in patients with CAP. With CURB-65, the AUROC for predicting 30-day mortality was 0.672 for patients aged ≥ 65 years^24^; when divided into two groups by age, the AUROCs were 0.64–0.74 and 0.59–0.64 for patients aged 65–84 years and ≥ 85 years, respectively^12–14^. This tendency of the discriminatory power to decline with increasing age was also observed in our study. There are two possible reasons for the underperformance of CURB-65 in an older population: 1) CURB-65 may have overestimated the severity in older patients, because it allocated one-point to the “age” variable uniformly to all patients aged ≥ 65 years; 2) the variables chosen for CURB-65 and their cut-off values were unable to encompass the clinical heterogeneity of older patients, including those with atypical clinical presentation and multiple underlying diseases. This is supported by a previous study showing that the multidimensional prognostic index, a multifaceted scoring system composed of social, educational, functional, and medical variables, was superior to PSI in predicting mortality in older patients with CAP^25^.

Our derivation cohort identified five factors independently associated with the 30-day mortality of CAP in older adults. This study revealed that confusion and azotemia, both of which were included in CURB-65, were risk factors for mortality in older adults with CAP, which is consistent with previous studies^12,14^.

Various studies have focused on the impact of the functional status of patients on the prognosis of pneumonia. The Barthel index, a clinical tool used to assess the ability to perform basic activities of daily living (ADL), has been shown to be an independent predictor of mortality in patients with CAP^26,27^. However, this index would be unsuitable in daily clinical practice because of the large number of variables to be evaluated and the complex calculation involved. Conversely, the Eastern Cooperative Oncology Group (ECOG) score, a simpler tool developed for functional evaluation in patients with cancer, was shown to be useful for predicting the prognosis of patients with CAP, even when limited to older adults^18,28^. In this study, an ECOG score of 4 (i.e., bedridden state) was a significant prognostic factor. Impaired ADL, especially in a bedridden state, may often be associated with decreased swallowing function and cough reflex impairment, suggesting that aspiration pneumonia, which has a high risk of mortality, is more likely to occur^29^.

Respiratory failure, including hypoxemia, is widely recognized as a poor prognostic factor for pneumonia, and several scoring systems have been established as severity assessment tools^4,28,30^. Moreover, SpO_2_, which can be easily measured with a pulse oximeter, has recently been reported to be useful for predicting the prognosis of patients with CAP^15,28,31^.

In this study, hypoalbuminemia was significantly associated with mortality in older patients with CAP. Several previous studies have demonstrated that hypoalbuminemia is a risk factor for poor prognosis in CAP using a cut-off value of 3.0 g/dL, which is in line with our study^32,33^. Hypoalbuminemia is associated with poorer outcomes, including increased complications and short-term and long-term mortality in critically ill patients. However, it remains uncertain whether the impact of hypoalbuminemia on outcome is a cause–effect relationship or whether hypoalbuminemia is a marker of critical condition^34^.

There are concerns about the adverse effects of hospitalization specific to older adults, including wasteful utilization of hospital resources and an increased risk of nosocomial infection. Moreover, hospitalization may result in a decline of ADL in older patients with acute-onset diseases, including CAP^35^. Approximately 30% of older patients admitted to hospitals also develop delirium^36^, which is considered a risk factor for in-hospital mortality in older patients with CAP^37^. Thus, identification of patients not necessarily requiring hospitalization is an important issue.

We propose the following management strategy for CAP in older adults based on the newly developed scoring system: patients with a score of 0 are at low risk of mortality (0.8% for 30-day mortality and 1.2% for in-hospital mortality) and are candidates for initial management with oral antimicrobial therapy as outpatients; patients with scores of 1 or 2 are at an intermediate risk of mortality (5.0% for 30-day mortality, and 8.1% for in-hospital mortality) and should be recommended for hospitalization; patients with a score ≥ 3 are at high risk of mortality (23.8% for 30-day mortality, and 30.5% for in-hospital mortality) and should be treated as severe pneumonia or terminal stage of life. Nonetheless, in clinical practice, social circumstances and patients’ wishes should also be taken into consideration when deciding how and where to treat older patients with CAP.

The derivation and validation cohorts in this study showed two characteristics of Japanese hospitals that reflects the aging society and features of the national healthcare system. First, the average length of hospitalization for CAP in both cohorts was about 3–4 weeks, which was longer than that in other countries. The possible reasons are as follows: 1) In Japan, it is common to extend the hospitalization period for acute illnesses even after the acute phase has passed, until the patient is transferred to an LTCF or discharged home. In this period, minor illnesses that may arise are taken care of. 2) The average age of both cohorts was > 80 years, and thus, several patients required prolonged hospitalization due to minor issues such as aspiration-related fever. Second, there were many differences in patient characteristics and clinical outcomes between the two cohorts. Ono Municipal Hospital, which recruited for the derivation cohort, was located in the suburbs and catered to older individuals, while all seven hospitals that recruited for the validation cohort were located in urban areas, focusing more on medical care for the acute phase of illness. Therefore, patients in the derivation cohort had a higher rate of LTCF use, longer period of hospitalization, and higher in-hospital mortality than those in the validation cohort. Further, the individual characteristics of each hospital may also have impacted the prevalence of underlying diseases and application of life-support measures. Though there were several differences between the two cohorts, the CHUBA scoring system was consistently validated, suggesting that it is widely applicable for the prognostication of CAP in older adults.

There are several potential limitations that should be acknowledged. First, our study sample may not reflect the full spectrum of the patient population because outpatients were not included in either the derivation or validation cohort. Second, the PSI score could not be calculated for some patients in the validation cohort because arterial blood gas analysis was not mandatory in this cohort. Therefore, the new system’s non-inferiority to PSI in the discriminatory ability for prognostic prediction could not be validated. However, considering the complexity of PSI, the successful development of an easy-to-use prognosis prediction system superior to CURB-65 remains significant.

In conclusion, the new scoring system CHUBA developed herein had better discriminative power in predicting mortality of older patients hospitalized with CAP than that of CURB-65. In the future, further investigation into varied cohorts is warranted to assess the generalizability of our findings.

## Supplementary Information

Supplementary Information.

## Data Availability

The datasets that support the findings of this study are available from the corresponding author on reasonable request.
