# Supplementary material for: Development and validation of a new scoring system for prognostic prediction of community-acquired pneumonia in older adults
Source: Sci Rep. 2021 Dec 13;11:23878. doi: 10.1038/s41598-021-03440-3 (PMC8668907; doi:10.1038/s41598-021-03440-3)
Supplement: Supplementary file 1 — Supplementary Information. [file 41598_2021_3440_MOESM1_ESM.pdf]

Supplementary Material

Development and validation of a new scoring system for prognostic prediction of community-acquired pneumonia in older adults

Masahiro Shirata, Isao Ito, Tadashi Ishida, Hiromasa Tachibana, Naoya Tanabe, Satoshi Konishi, Issei Oi, Nobuyoshi Hamao, Kensuke Nishioka, Hisako Matsumoto, Yoshiro Yasutomo, Seizo Kadowaki, Hisashi Ohnishi, Hiromi Tomioka, Takashi Nishimura, Yoshinori Hasegawa, Atsushi Nakagawa, Toyohiro Hirai

Supplementary Figure 1.

(A) Derivation cohort (age 65–84 years)

i) 30-day mortality

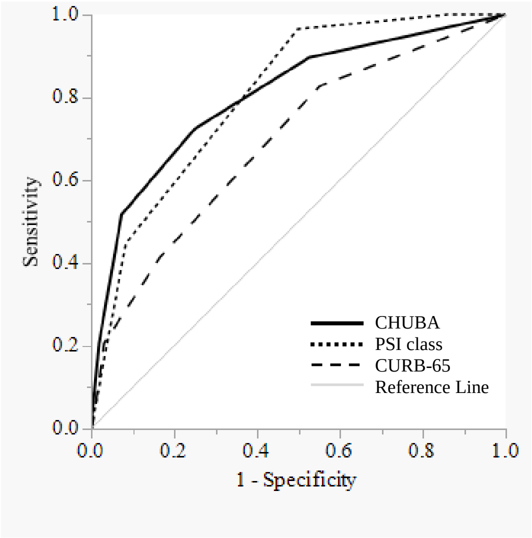

	AUROC (95% CI)	*P* value
1) CHUBA	0.805 (0.702–0.878)	1 vs 2: 0.953
2) PSI class	0.807 (0.740–0.860)	1 vs 3: 0.017
3) CURB-65	0.694 (0.590–0.782)	

ii) In-hospital mortality

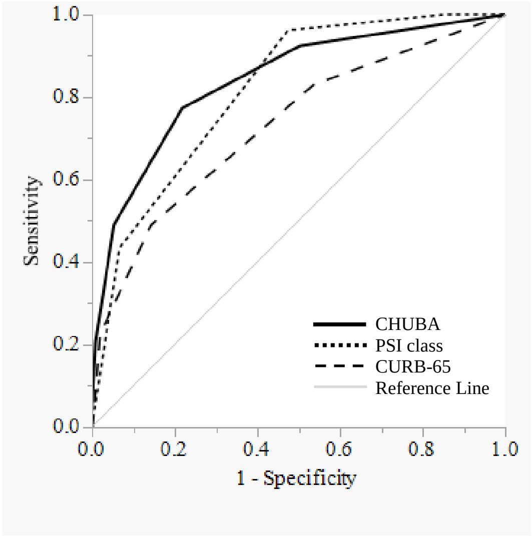

	AUROC (95% CI)	*P* value
1) CHUBA	0.841 (0.774–0.891)	1 vs 2: 0.414
2) PSI class	0.818 (0.768–0.859)	1 vs 3: 0.004
3) CURB-65	0.730 (0.651–0.797)	

(B) Derivation cohort (age ≥ 85 years)

i) 30-day mortality

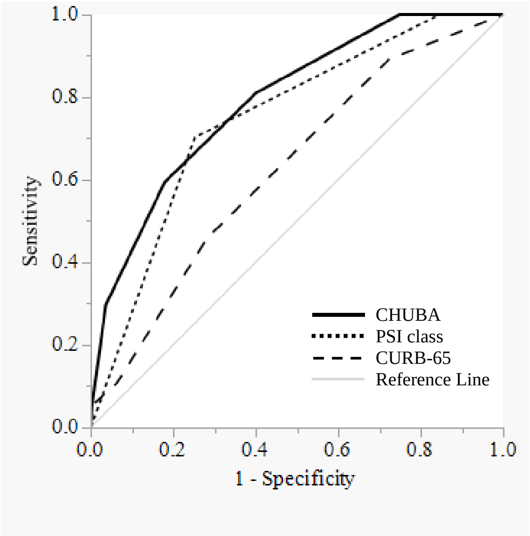

	AUROC (95% CI)	*P* value
1) CHUBA	0.792 (0.713–0.853)	1 vs 2: 0.130
2) PSI class	0.748 (0.674–0.810)	1 vs 3: <0.001
3) CURB-65	0.627 (0.538–0.708)	

ii) In-hospital mortality

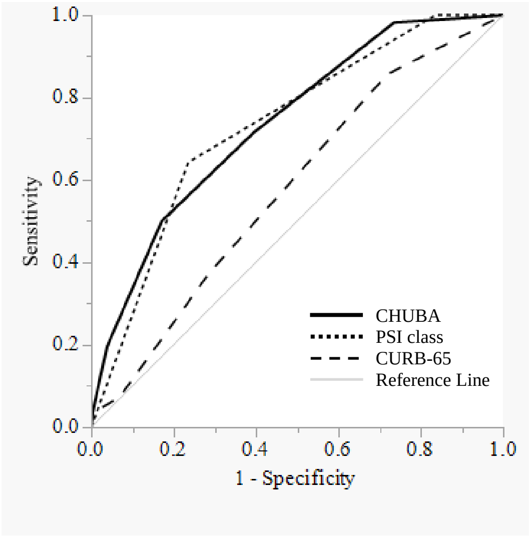

	AUROC (95% CI)	*P* value
1) CHUBA	0.738 (0.667–0.798)	1 vs 2: 0.878
2) PSI class	0.733 (0.670–0.788)	1 vs 3: <0.001
3) CURB-65	0.579 (0.506–0.650)	

Supplementary Figure 2.

Derivation cohort

i) 30-day mortality

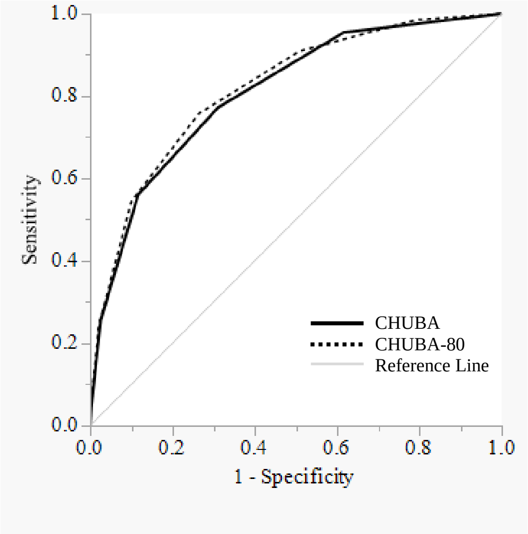

	AUROC (95% CI)	*P* value
1) CHUBA	0.809 (0.751–0.856)	0.200
2) CHUBA-80	0.819 (0.762–0.866)	

ii) In-hospital mortality

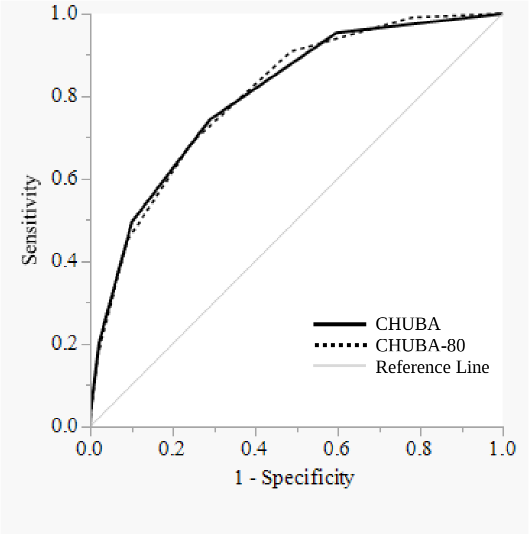

	AUROC (95% CI)	*P* value
1) CHUBA	0.802 (0.757–0.840)	0.896
2) CHUBA-80	0.803 (0.759–0.840)	

Supplementary Figure 3.

(A) Validation cohort (age 65–84 years)

i) 30-day mortality

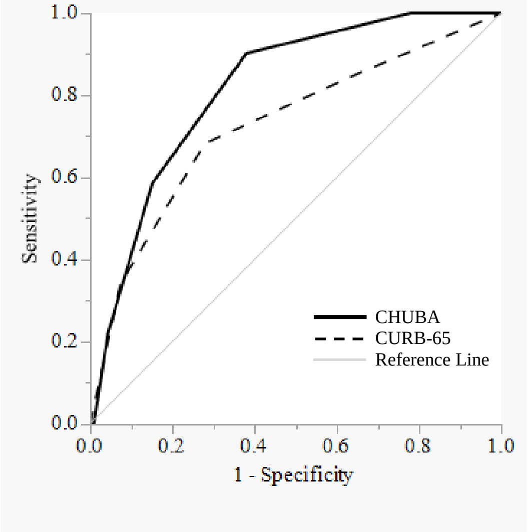

	AUROC (95% CI)	*P* value
1) CHUBA	0.818 (0.759–0.865)	0.028
2) CURB-65	0.726 (0.633–0.803)	

ii) In-hospital mortality

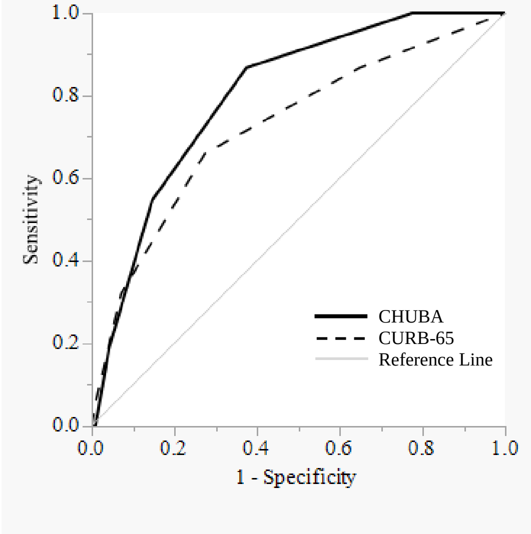

	AUROC (95% CI)	*P* value
1) CHUBA	0.802 (0.747–0.847)	0.026
2) CURB-65	0.726 (0.647–0.793)	

(B) Validation cohort (age ≥ 85 years)

i) 30-day mortality

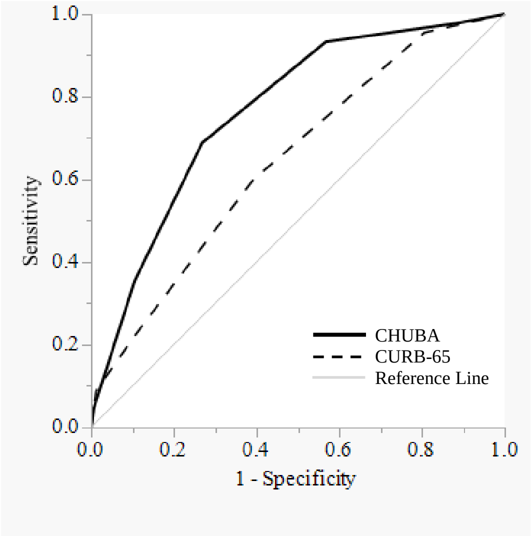

	AUROC (95% CI)	*P* value
1) CHUBA	0.765 (0.691–0.825)	0.004
2) CURB-65	0.644 (0.562–0.718)	

ii) In-hospital mortality

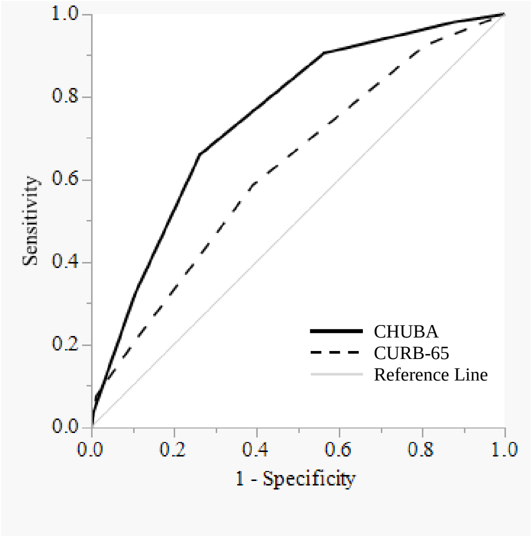

	AUROC (95% CI)	*P* value
1) CHUBA	0.748 (0.678–0.807)	0.004
2) CURB-65	0.627 (0.548–0.699)	

Figure Legends

Supplementary Figure 1.

Comparisons of the discriminatory power of CURB-65 (confusion, urea, respiratory rate, blood pressure, age ≥65), pneumonia severity index (PSI) class, and CHUBA (confusion, hypoxemia, urea, bedridden, albumin) for prediction of mortality among older patients aged 65–84 years and those aged >85 years, hospitalized with community-acquired pneumonia in the derivation cohort.

(A) The areas under the receiver operating characteristic (AUROCs) of CHUBA for prediction of both 30-day mortality and in-hospital mortality among older patients aged 65–84 years were significantly higher than those of CURB-65 (*P*=0.017 and *P*=0.004, respectively), and were statistically equivalent to those of PSI class (*P*=0.953 and *P*=0.414, respectively).

(B) AUROCs of CHUBA for prediction of both 30-day mortality and in-hospital mortality among those aged >85 years were significantly higher than those of CURB-65 (both *P* <0.001), and were statistically equivalent to those of PSI class (*P*=0.130 and *P*=0.878, respectively).

Supplementary Figure 2.

Comparisons of the discriminatory power of CHUBA (confusion, hypoxemia, urea, bedridden, albumin) and CHUBA-80 (confusion, hypoxemia, urea, bedridden, albumin, age ≥80) for predicting mortality among older patients hospitalized with a diagnosis of community-acquired pneumonia in the derivation cohort.

The areas under the receiver operating characteristic (AUROCs) of CHUBA-80 for prediction of both 30-day mortality and in-hospital mortality were significantly equivalent to those of CHUBA (*P*=0.200 and *P*=0.896, respectively).

Supplementary Figure 3.

Comparisons of the discriminatory power of CURB-65 (confusion, urea, respiratory rate, blood pressure, age ≥65), pneumonia severity index (PSI) class, and CHUBA (confusion, hypoxemia, urea, bedridden, albumin) for prediction of mortality among older patients aged 65–84 years and those aged >85 years, hospitalized with community-acquired pneumonia in the validation cohort.

(A) The areas under the receiver operating characteristic (AUROCs) of CHUBA for prediction of both 30-day mortality and in-hospital mortality among older patients aged 65–84 years were significantly higher than those of CURB-65 (*P*=0.028 and *P*=0.026, respectively).

(B) AUROCs of CHUBA for prediction of both 30-day mortality and in-hospital mortality among those aged >85 years were significantly higher than those of CURB-65 (both *P*=0.004).
